# Melanoma with osteocartilaginous differentiation^☆^

**DOI:** 10.1016/j.abd.2022.04.004

**Published:** 2023-01-02

**Authors:** Bennett Barroso de Carvalho, Diogo Batista dos Santos Medeiros

**Affiliations:** Division of Pathology, Hospital de Base do Distrito Federal, Brasília, DF, Brazil

*Dear Editor,*

Melanoma is a potentially lethal skin neoplasm that requires an accurate diagnosis to ensure that the patient will receive optimal treatment. Despite the advent of large-scale, high-resolution genomics, histopathological analysis remains the reference for diagnosis and the primary classification tool in correlation with clinical characteristics^1^.

Melanoma can exhibit a variety of histopathological patterns, mimicking other malignant tumors. Based on their histopathological characteristics, several morphological subtypes have been described in addition to the classical forms, such as polypoid, verrucous, desmoplastic, myxoid, balloon-cell, rhabdoid, amelanotic, and spitzoid melanoma, among others^2^. It may also rarely show heterologous differentiation patterns, including fibroblastic, smooth muscle, rhabdomyoblastic, osteocartilaginous, Schwannian, and ganglionic. Osteocartilaginous differentiation is one of the most uncommon patterns^3^, ^4^.

Osteocartilaginous differentiation is characterized by the formation of osteoid, bone, and cartilaginous tissue by the neoplastic cells. Its rarity and overlapping characteristics with those of other neoplasms make it difficult to diagnose, especially in small biopsies or when clinical/radiological information is limited. It may be confused with osteosarcomas or chondrosarcomas^5^.

The mechanisms responsible for heterologous differentiation are not well understood. This report describes a case of melanoma with osteocartilaginous differentiation that developed on the distal region of the left upper limb.

In August 2019, a 52-year-old woman came to the Orthopedics outpatient clinic with a history of a darkened lesion in the subungual region of the left thumb, with an evolution of approximately three years. On physical examination, edema, local phlogosis, and signs suggestive of fungal infection were observed. She underwent specific treatment for the infection but did not show a complete response.

The subungual lesion was submitted to an incisional biopsy, and the histopathological and immunohistochemical study confirmed the diagnosis of melanoma. The patient was then referred to the Oncological Surgery service for specific treatment.

In November of the same year, after evaluation, she underwent amputation of the affected finger. Histopathological analysis confirmed the diagnosis of nodular melanoma, with the presence of lymphovascular invasion and a proliferative index of 17 mitoses/mm^2^. After surgery, she had regular follow-up consultations at the Clinical Oncology department. After eight months, tumor growth was observed on the surgical scar. Computed tomography assessment showed a lesion suggestive of recurrence.

She underwent surgical resection once again, with en bloc removal of the lesion. Histopathological analysis of the specimen showed local recurrence of the melanoma, with the presence of vascular and neural invasion, in addition to compromised surgical margins. Follow-up imaging studies demonstrated the presence of axillary involvement and the presence of pulmonary lesions suggestive of metastasis.

The patient was referred for adjuvant local and axillary radiotherapy in February 2021. She also started therapy with Dacarbazine and immunotherapy with Pembrolizumab in April 2021. In the same month, she had a new tumor growth in the area of ​​the previous resection. Computed tomography showed extensive expansive/infiltrative soft tissue lesions in the distal region of the left arm and hand, and palliative surgery was chosen, with surgical disarticulation at the elbow and left axillary lymphadenectomy, performed in July 2021.

Macroscopically, the surgical specimen showed a large tumor in the distal region of the left upper limb (Fig. 1).

**Figure 1:**
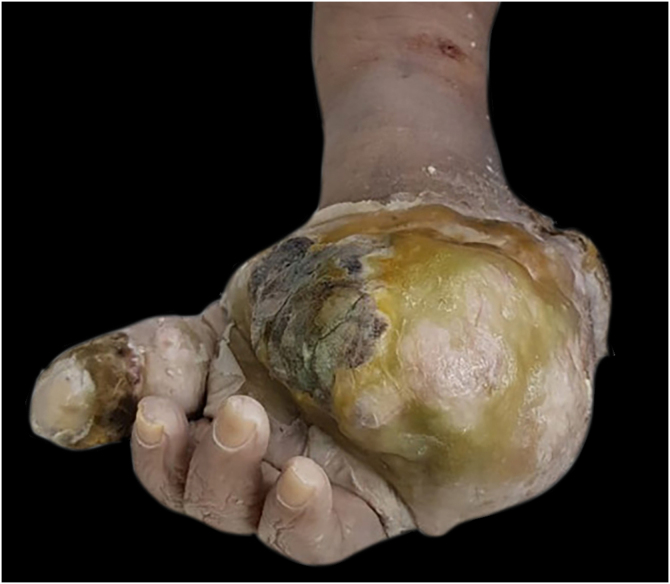
Tumor in the distal region of the left upper limb.

Histopathology showed the presence of melanoma with areas of heterologous osteocartilaginous differentiation (Figure 2, Figure 3, Figure 4), invading soft tissues and bone, with free surgical margins. The diagnosis of melanoma was confirmed by immunoreactivity with SOX10 and HMB45 (Fig. 5). Axillary dissection showed metastatic melanoma in a left axillary lymph node. Molecular screening for the V600E mutation in the BRAF gene was negative.

**Figure 2:**
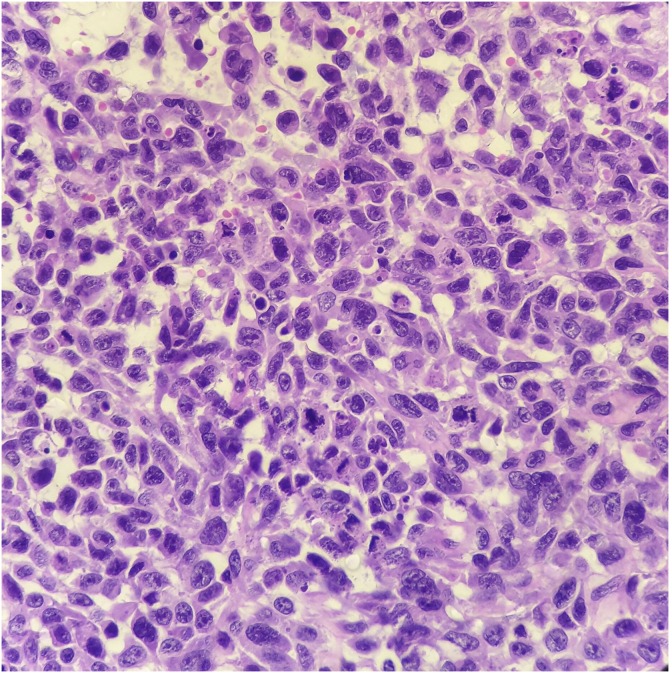
Melanoma cells showing atypia, pleomorphism, evident nucleoli, and frequent mitotic figures (Hematoxylin & eosin, ×400).

**Figure 3:**
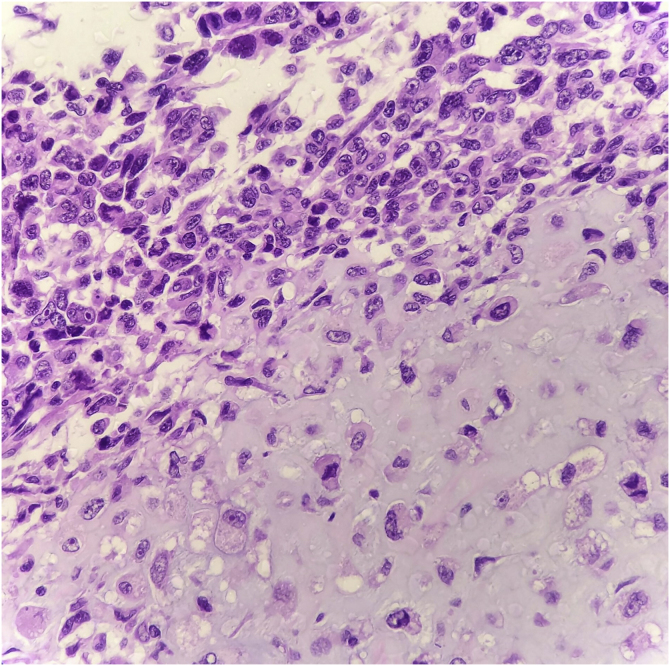
Area of transition into heterologous chondroid differentiation (Hematoxylin & eosin, ×400).

**Figure 4:**
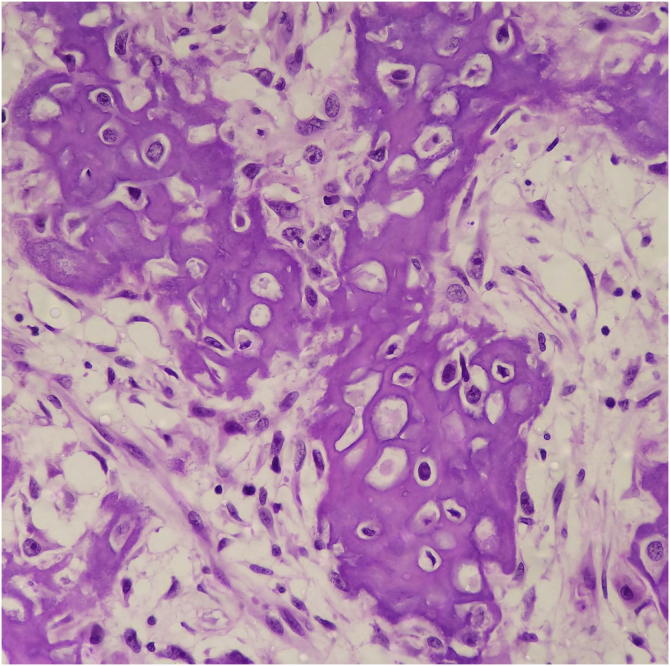
Area of transition into heterologous osteoid differentiation (Hematoxylin & eosin, ×400).

**Figure 5:**
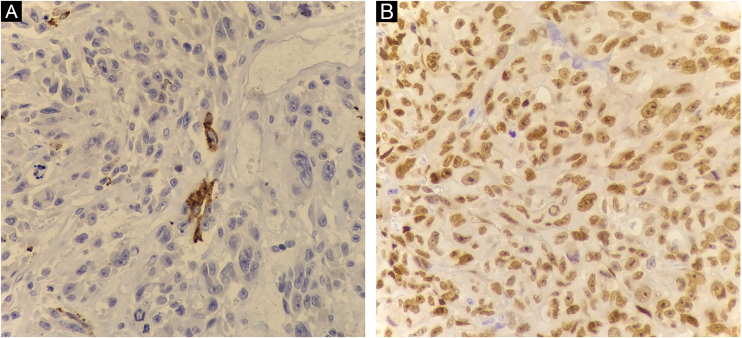
(A) Immunoreactivity with HMB45 (×400). (B) Immunoreactivity with SOX10 (×400).

Due to advanced and metastatic disease, the patient remained hospitalized, progressing with clinical worsening, signs of respiratory distress, and hemodynamic instability. With no possibility of disease-modifying therapy, she started exclusive palliative care, passing away in October 2021.

Osteocartilaginous differentiation in melanoma was first observed over 45 years ago^6^, but fewer than 40 reports have been published since then^7^, ^8^.

Osteoid, chondroid and mixed patterns have been described as appearing in predominantly acral and subungual sites. However, osteocartilaginous differentiation is not restricted to these regions, as it has been previously described in mucosal lesions, as well as in skin that is chronically or intermittently exposed to the sun^9^.

The mechanism for the formation of the osteoid/chondroid matrix in these melanomas is unknown. Many are associated with a history of previous local trauma and the osteoid/chondroid matrix may be a form of host response to the lesion^10^. Another hypothesis is that osteoid/chondroid differentiation is a result of reactive responses induced by the melanoma in the surrounding stromal fibroblasts. However, there is often a lack of stromal cells in the matrix areas, which may arise from the neoplastic cells themselves^11^.

Hypothetical intrinsic melanoma factors that could drive the production of this matrix include the expression of the “Melanoma inhibitory activity (MIA)” and “Runt-related transcription factor 2” (RUNX2) genes, transcriptional regulators of chondrogenesis and osteogenesis^10^.

Although these genes can be expressed by most melanomas, studies have already shown that MIA and RUNX2 require the expression of other cofactors to drive osteoid/chondroid differentiation, and the lack of these genes may explain the rarity of osteocartilaginous melanoma despite frequent gene expression^8^.

The diagnosis of melanoma can be supported by the presence of an “*in situ*” component, the presence of melanin pigment, or immunoreactivity with markers such as S100, Melan-A, HMB45, or SOX10^1^. In melanomas with cartilaginous differentiation, the use of S100 is limited, as cartilaginous tissues and chondrosarcomas also express positivity^5^.

In the molecular analysis, the presence of the V600E mutation in the BRAF gene can also support the diagnosis of melanoma, in addition to being crucial for planning its treatment^5^.

It is not yet entirely clear whether the osteocartilaginous melanoma harbors only mutations and differentiation markers typical of melanoma or of the osteocartilaginous lesions it mimics as well^8^.

Based on the literature review, the authors highlight the importance of applying an immunohistochemical panel with the inclusion of melanocytic differentiation markers in cases of neoplasms with osteoid/chondroid differentiation, mainly in acral lesions, and of including melanoma with heterologous differentiation among the differential diagnoses. Due to the rarity of the case, the development of guidelines for early diagnosis, management and prognosis for this cancer variant remains challenging.

## Financial support

None declared.

## Authors' contributions

Bennett Barroso de Carvalho: drafting and editing of the manuscript or critical review of important intellectual content; critical review of the literature.

Diogo Batista dos Santos Medeiros: Effective participation in research orientation; drafting and editing of the manuscript or critical review of important intellectual content; approval of the final version of the manuscript.

## Conflicts of interest

None declared.

## References

[1] International Agency for Research on Cancer (2018).

[2] Rongioletti F., Smoller B.R. (2005). Unusual histological variants of cutaneous malignant melanoma with some clinical and possible prognostic correlations. J Cutan Pathol.

[3] Banerjee S.S., Harris M. (2000). Morphological and immunophenotypic variations in malignant melanoma. Histopathology.

[4] Banerjee S.S., Coyne J.D., Menasce L.P., Lobo C.J., Hirsch P.J. (1998). Diagnostic lessons of mucosal melanoma with osteocartilaginous differentiation. Histopathology.

[5] Berro J., Halim N.A., Khaled C., Assi H.I. (2019). Malignant melanoma with metaplastic cartilaginous transdifferentiation: A case report. J Cutan Pathol.

[6] Pluta J., Malska-Waniewska I. (1974). Osseous changes in malignant melanoma. Nowotwory.

[7] Murali R., McCarthy S.W., Bothman J., Cachia A., Janarthanan P., Sharma R. (2010). Melanoma exhibiting cartilaginous differentiation. Histopathology.

[8] Gallagher S.J., Bailey T., Rawson R.V., Mahar A.M., Thompson J.F., Long G.V. (2021). Melanoma with osseous or chondroid differentiation: a report of eight cases including SATB2 expression and mutation analysis. Pathology.

[9] Ali A.M., Wang W.L., Lazar A.J. (2018). Primary chondro-osseous melanoma (chondrosarcomatous and osteosarcomatous melanoma). J Cutan Pathol.

[10] Parente J.D., da Silva Labareda J.M.P., Bártolo E.A.F.L.F., Santos M.F.S.P.F., do Vale E.M.S. (2013). Melanoma cartilagíneo: Caso clínico e revisão da literature. An Bras Dermatol.

[11] Savant D., Kenan S., Kenan S., Kahn L. (2018). Osteogenic melanoma: report of a case mimicking osteosarcoma and review of the literature. Skeletal Radiol.

